# Screening and Validation of a Carvacrol-Targeting Viability-Regulating Protein, SLC6A3, in Liver Hepatocellular Carcinoma

**DOI:** 10.1155/2022/3736104

**Published:** 2022-03-30

**Authors:** Xieling Yin, Hongjian Chen, Shi Chen, Suqing Zhang

**Affiliations:** Department of Hepatobiliary and Pancreatic Surgery, Tumor Hospital Affiliated To Nantong University, China

## Abstract

**Background:**

Liver hepatocellular carcinoma (LIHC) is the second leading cause of tumor-related death in the world. Carvacrol was also found to inhibit multiple cancer types. Here, we proposed that Carvacrol inhibited LIHC.

**Methods:**

We used MTT assay to determine the inhibition of Carvacrol on LIHC cells. BATMAN-TCM was used to predict targets of Carvacrol. These targets were further screened by their survival association and expression in cancer using TCGA data. The bioinformatic screened candidates were further validated in *in vitro* experiments and clinical samples. Finally, docking models of the interaction of Carvacrol and target protein were conducted.

**Results:**

Carvacrol inhibited the viability of LIHC cell lines. 40 target genes of Carvacrol were predicted, 8 of them associated with survival. 4 genes were found differentially expressed in LIHC vs. normal liver. Among these genes, the expression of SLC6A3 and SCN4A was found affected by Carvacrol in LIHC cells, but only SLC6A3 correlated with the viability inhibition of Carvacrol on LIHC cell lines. A docking model of the interaction of Carvacrol and SLC6A3 was established with a good binding affinity. SLC6A3 knockdown and expression revealed that SLC6A3 promoted the viability of LIHC cells.

**Conclusion:**

Carvacrol inhibited the viability of LIHC cells by downregulating SLC6A3.

## 1. Introduction

Liver hepatocellular carcinoma (LIHC), the most common type of primary liver cancer, is the second leading cause of tumor-related death in the world [1]. There are 905.7 new liver cancer cases and 830.2 death from liver cancer per 100 000 people in 2020 in the world [2]. According to 2021 Cancer Statistics, liver and intrahepatic bile duct cancer accounted for 42,230 new cases and 30,230 cancer death in 2020 [3]. Over the past several decades, the study in LIHC management has made a limited improvement, and the outcome of LIHC treatment remains undesirable. The earliest FDA-approved anti-LIHC agents for late-stage LIHC treatment included sorafenib regorafenib and lenvatinib. These drugs are all subjected to low response rates, thus, further progress is required for their application in clinics [4–6]. Traditional medicine and naturally occurring compounds have been being studied intensively for their applications in the management of human diseases [7–12]. Traditional medicine has been applied wildly in clinical cancer treatment as complementary and supplementary medicine, especially in China, Korea, and Japan where traditional medicine is part of the healthcare system [13]. However, so far, there is no natural medication that has been approved by the FDA for the treatment of liver hepatocellular carcinoma. Hence, more understanding of the effect of natural medication on this type of cancer is required for future clinical applications. Carvacrol, a phenol that is a natural monoterpene derivative of cymene, has been used for antifungal, antiviral, treatment for cancer, and regulation of inflammatory activities [14]. Carvacrol was first discovered as a nonspecific inhibitor for the transient receptor potential melastatin-like 7 channel (TRPM7) [15], which is a potential target for cancers [16, 17]. Carvacrol was also found to inhibit multiple cancer type, including breast cancer cell lines [18, 19], cervical cancer [20, 21], ovarian cancer [22], prostate cancer [23–28], colon cancer [29, 30], lung cancer [31], and oral cancer [32]. However, so far, few studies have reported its effect on LIHC. An *in vitro* study has reported the antiproliferative and proapoptotic effect of Carvacrol on human hepatocellular carcinoma cell line HepG-2 [33]. Our hospital has applied Carvacrol in-hospital preparations for LIHC patients as supplementary medicine and has achieved desirable outcomes in many cases. Although no systematic data regarding this issue has been published, we proposed that Carvacrol might potentially inhibit LIHC. This study provided preclinical evidence to support the clinical application of Carvacrol for LIHC. In addition, although many studies reported potential targets and mechanisms of Carvacrol, pharmacological targets of Carvacrol remain largely unidentified. In this study, we screened pharmacological targets of Carvacrol in LIHC. This study identified a potential target of Carvacrol and is conducive to the optimization of the clinical application of Carvacrol in LIHC treatment.

## 2. Results

### 2.1. The Effect of Carvacrol on the Viability of LIHC Cells

**Figure 1:**
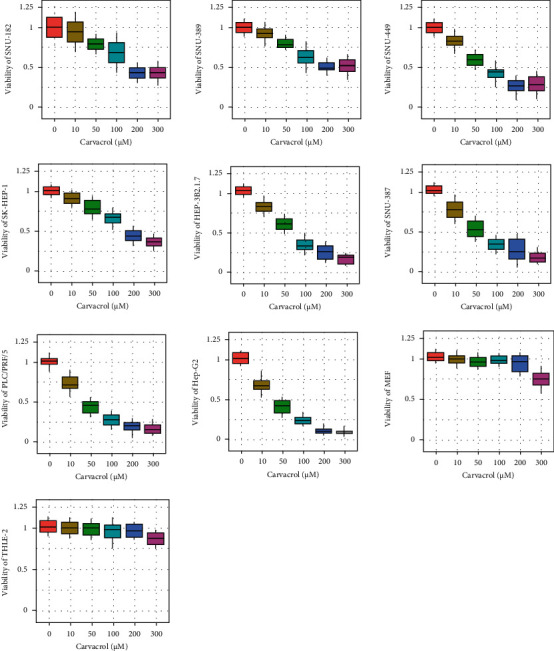
The effect of Carvacrol on the viability of eight LIHC cell lines, including SNU-182, SNU-398, SNU-449, SK-HEP-1, HEP-3B2.1-7, SNU-387, PLC/PRF/5, and Hep-G2. Primary cells MEF and THLE-2 were used as normal control. Cells were exposed to 10-300 *μ*M Carvacrol for 24 hours, and the viability was determined using MTT assay.

In this study, we first determined the effect of Carvacrol on the viability of eight LIHC cell lines, including SNU-182, SNU-398, SNU-449, SK-HEP-1, HEP-3B2.1-7, SNU-387, PLC/PRF/5, and Hep-G2. Results showed that the viability of all of these cell lines was inhibited by Carvacrol at 10-300 *μ*M with different sensitivity. The most sensitive cell line was Hep-G2 with an inhibition rate of up to 90% at 300 *μ*M Carvacrol. The least sensitive cell lines were SNU-182 and SNU-389, both of which with an inhibition rate of 40% at 300 *μ*M Carvacrol. In addition, we used primary cells MEF and THLE-2 as controls to demonstrate the cancer specificity of Carvacrol. Results showed that the viability of MEF and THLE-2 only significantly decreased at 300 *μ*M (Figure 1). Thus, we suggest that Carvacrol can inhibit the viability of LIHC cells. 200 *μ*M was used in the subsequent study as it did not significantly affect the control cells. The viability IC50 values of Carvacrol were displayed in Table 1.

### 2.2. Prediction of Targets of Carvacrol in LIHC

**Figure 2:**
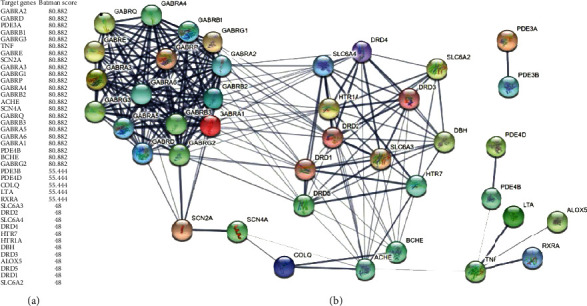
Identification of candidate targets of Carvacrol in LIHC. (a) Carvacrol target genes and their batman scores. (b) Protein-protein interaction network of Carvacrol target genes.

In this study, we first used BATMAN-TCM to predict potential targets of Carvacrol. For each compositive compound, the predicted candidate targets whose scores given by the target prediction method exceed a cutoff batman score of >40 were considered as the potential targets of Carvacrol and were presented (Figure 2(a)). BATMAN-TCM used a similarity-based method to predict potential targets of Carvacrol, the core idea of which was to rank potential drug-target interactions based on their similarity to the known drug-target interactions [34]. The batman score was calculated as the product of the drug similarity score and the target similarity score in the known drug-target interactions. Using this algorithm, we obtain 40 target genes of Carvacrol. These target genes were further constructed into a protein-protein interaction network using the Search Tool for the Retrieval of Interacting Genes/Proteins (STRING) (Figure 2(b)).

### 2.3. Survival Association of Targets of Carvacrol in LIHC

**Figure 3:**
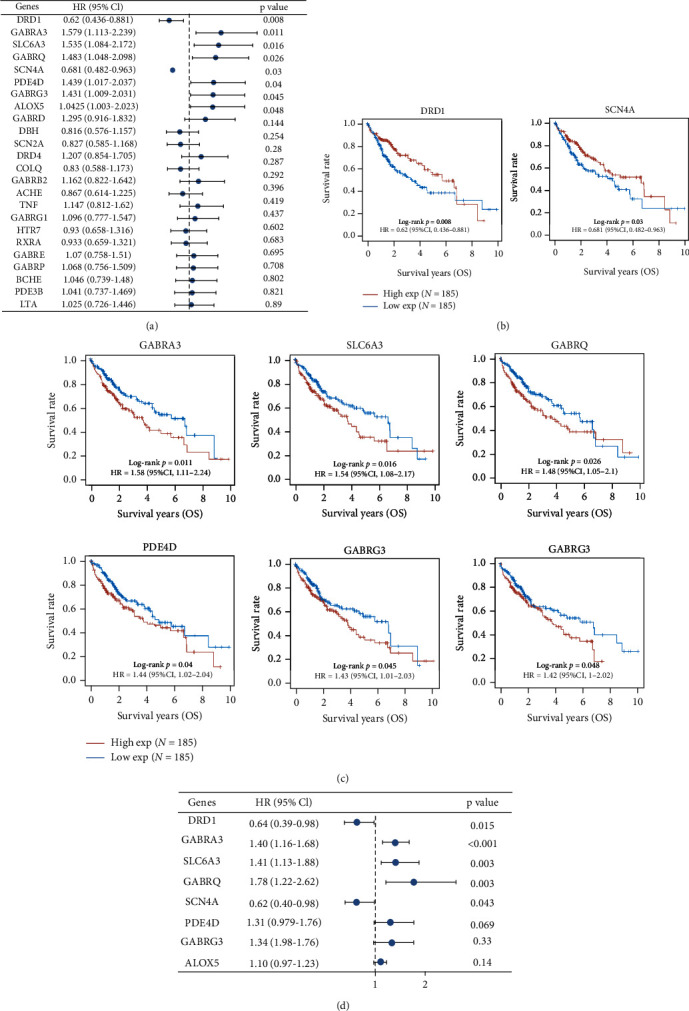
Survival association of candidate targets of Carvacrol in LIHC. (a) Hazard ratio of candidate target genes using log-rank analysis. Only 24 candidate target genes with the lowest *p* value were shown. (b) MK-plots of protective targets of Carvacrol in LIHC. (c) MK-plots of hazard targets of Carvacrol in LIHC. (d) Univariate Cox regression analysis of overall survival and target genes.

To identify potential effective targets of Carvacrol in LIHC, we screened the association of all these targets using log-rank analysis using TCGA LIHC cohort. Results showed that eight target genes were significantly associated with the overall, including two protective genes, DRD1 (HR 0.436-0.881) and SCN4A (HR 0.482-0.963), and six hazard genes, GABRA3 (HR 1.113-2.239), SLC6A3 (HR 1.084-2.172), GABRQ (HR 1.048-2.098), PDE4D (HR 1.017-2.037), GABRG3 (HR 1.009-2.031), and ALOX5 (HR 1.003-2.023) (Figure 3(a)). These eight genes were identified as potential effective targets of Carvacrol in LIHC and were further screened in the subsequent study. In addition, we further plotted the MK curves for these significant genes (Figures 3(b) and 3(c)) and conducted a univariate Cox regression analysis. Results showed that GABRA3, SLC6A3, and GABRQ were positively associated with overall survival with a hazard ratio of 1.40 (1.16-1.68), 1.41 (1.13-1.88), and 1.78 (1.22-2.62), respectively, while DRD1 and SCN4A were negatively associated with overall survival with a hazard ratio of 0.64 (0.39-0.98) and 0.62 (0.40-0.98). The Cox regression analysis further confirmed the survival association of GABRA3, SLC6A3, GABRQ, DRD1, and SCN4A (Figure 3(d)).

### 2.4. The Overexpression of Target Genes in LIHC

**Figure 4:**
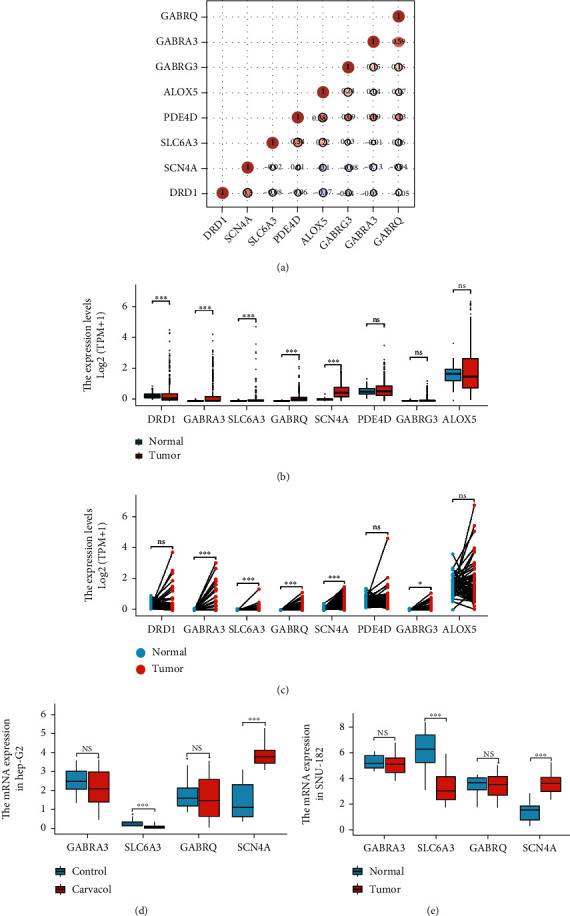
The expression of Carvacrol candidate target genes in LIHC. (a) The correlations of expression of Carvacrol candidate target genes in LIHC. (b) The expression level of Carvacrol candidate target genes in LIHC and liver tissue from TCGA and GTEx data. (c) The expression level of Carvacrol candidate target genes in LIHC paired cancer-noncancer samples from TCGA data. (d) The effect of Carvacrol on mRNA expression of potential targets in LIHC cell line Hep-G2. (e) The effect of Carvacrol on mRNA expression of potential targets in LIHC cell line SNU182. Cells were exposed to 200 *μ*M Carvacrol for 24 hours before the assay.

To identify the cancer-specific targets in LIHC, we compared the expression of these target genes in cancer vs. noncancer tissues using the TCGA LIHC cohort. First, we analyzed the coexpression of these target genes. The most correlated genes pair was GABRA3 and GABRAQ with a coefficient factor of 0.59 (Figure 4(a)). These data provide potential interactions of these genes. Then, we compared the expression of these target genes in cancer vs. noncancer tissues using TCGA LIHC cohort with GETx liver tissue cohort. Results showed that expressions of DRD1, GABRA3, SLC6A3, GABRQ, and SCN4A in cancer were significantly higher than those in noncancer tissues (Figure 4(b)). In addition, we further analyzed the expression level of these target genes in LIHC paired samples from TCGA data. Cancer and noncancer data from the same patients were compared and analyzed by paired *t*-test [35–36]. Results showed that GABRA3, SLC6A3, GABRQ, SCN4A, and GABRG3 were overexpressed in cancer compared with normal liver tissues (Figure 4(c)). Therefore, based on these results, we suggested that GABRA3, SLC6A3, GABRQ, and SCN4A might be cancer-specific targets of Carvacrol which were further screened in the subsequent study.

### 2.5. Effect of Carvacrol on Expressions of Target Genes in LIHC Cells

To further screen the target genes, we determined the effect of Carvacrol on gene expression of these target genes in two LIHC cell lines Hep-G2 and SNU-182 using QPCR. Cells were exposed to 200 *μ*M Carvacrol for 24 hours before the assay. Results revealed that in Hep-G2 cells, the expression of SLC6A3 was significantly increased by Carvacrol, the expression of SCN4A was significantly decreased by Carvacrol, and the expressions of GABRA3 and GABRQ were not affected (Figure 4(d)). In addition, in SNU-182, the expression of SLC6A3 was significantly increased by Carvacrol, the expression of SCN4A was significantly decreased by Carvacrol, and the expressions of GABRA3 and GABRQ were not affected (Figure 4(e)). Thus, we suggested that SLC6A3 and SCN4A might be direct targets of Carvacrol and will be analyzed in the subsequent study.

### 2.6. Correlation of Target Expression and Sensitivity to Carvacrol

**Figure 5:**
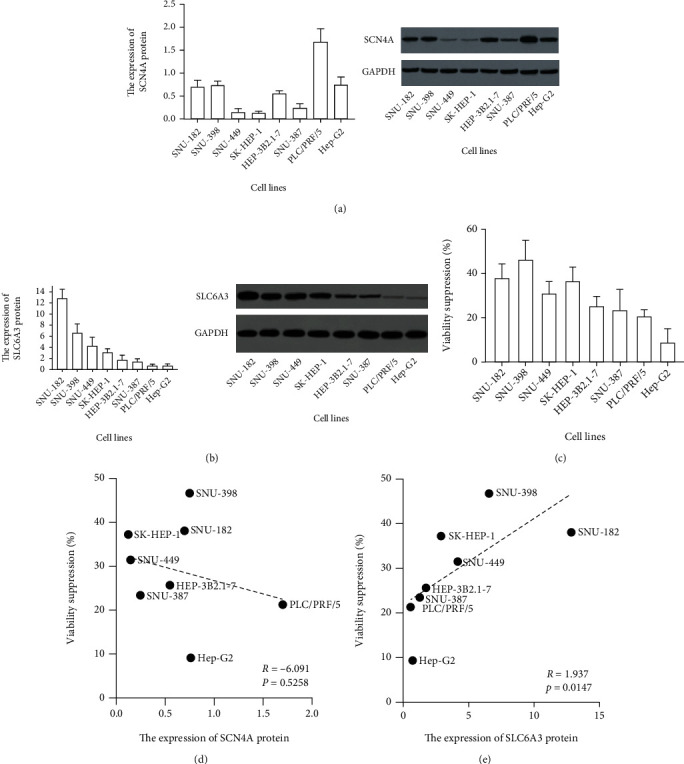
Correlation of SCN4A and SLC6A3 expression and LIHC cell viability suppression by Carvacrol. (a) The protein expression level of SCN4A in LIHC cell lines and image of the western blotting. (b) The protein expression level of SLC6A3 in LIHC cell lines and image of the western blotting. (c) The cell viability suppression rate of LIHC cell lines after 24-hour exposure to 200 *μ*M Carvacrol. (d) Correlation of SCN4A expression and LIHC cell viability suppression. (e) Correlation of SLC6A3 expression and LIHC cell viability suppression.

In the subsequent study, we determined the protein levels of SLC6A3 and SCN4A in eight LIHC cell lines, including SNU-182, SNU-398, SNU-449, SK-HEP-1, HEP-3B2.1-7, SNU-387, PLC/PRF/5, and Hep-G2. Results showed that PLC/PRF/5 expressed the highest level of SCN4A. SNU-449 and SK-HEP-1 expressed the lowest level of SCN4A (Figure 5(a)). Besides, SNU-182 expressed the highest level of SLC6A3. PLC/PRF/5 and Hep-G2 expressed the lowest level of SLC6A3 (Figure 5(b)). Furthermore, we determined the viability of these cell lines with or without the 24-hour exposure of 200 *μ*M Carvacrol and calculated the viability suppression rate of these cell lines. Results showed that SNU-182 and SNU-398 had the highest viability suppression rate while Hep-G2 had the lowest viability suppression rate after the exposure to Carvacrol (Figure 5(c)). We also further calculated the correlation of expression of SLC6A3 and SCN4A in these cell lines and their sensitivity to Carvacrol. Results showed that the expression of SCN4A in these cell lines was not correlated with their sensitivity (Figure 5(d)), but the expression of SLC6A3 in these cell lines was significantly correlated with their viability suppression rate with a coefficient of 1.937 (Figure 5(e)). These results indicated that SLC6A3 might mediate the effect of Carvacrol.

### 2.7. Effect of Carvacrol on Expressions of Target Genes in LIHC Tissue

**Figure 6:**
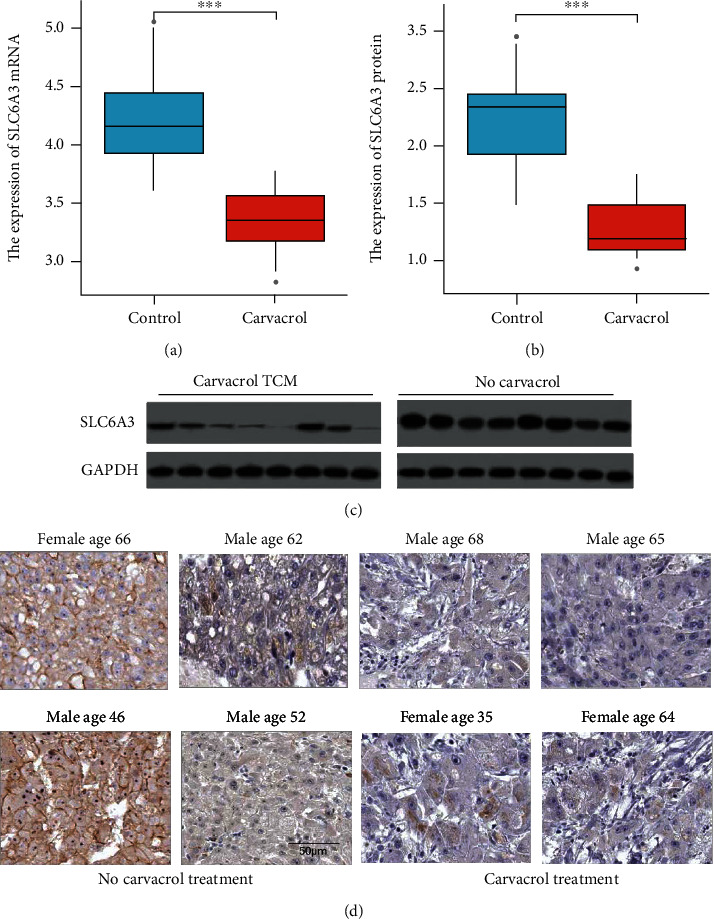
The effect of Carvacrol on the expression of SLC6A3 in LIHC. (a) The mRNA expression of SLC6A3 in LIHC samples was collected from patients with or without Carvacrol treatment. (b) The protein expression of SLC6A3 in LIHC samples was collected from patients with or without Carvacrol treatment. (c) Representative images of the western blotting. (d) Representative images of protein staining of SLC6A3 in LIHC samples collected from patients with or without Carvacrol treatment.

To validate the regulatory effect of Carvacrol on the expression of SLC6A3, we collected LIHC tissues from 18 patients with Carvacrol treatment and 18 patients without Carvacrol treatment. The prescription of Carvacrol treatment depended on the clinical condition of the patients, and the Carvacrol was applied as a supplementary therapy for patients. Hence, the dose of Carvacrol might vary from patient to patient. Nevertheless, the comparison of samples from patients with or without Carvacrol treatment can provide a hint at the effect of Carvacrol. Results showed that cancer tissue from patients with Carvacrol treatment expressed significantly lower SLC6A3 at both mRNA and protein levels compared with cancer tissue from patients without Carvacrol treatment (Figures 6(a)–6(c)). The protein staining of SLC6A3 in LIHC samples collected from patients with or without Carvacrol treatment further confirmed that SLC6A3 expression was downregulated by Carvacrol treatment (Figure 6(d)).

### 2.8. Binding Potential of Carvacrol to SLC6A3

**Figure 7:**
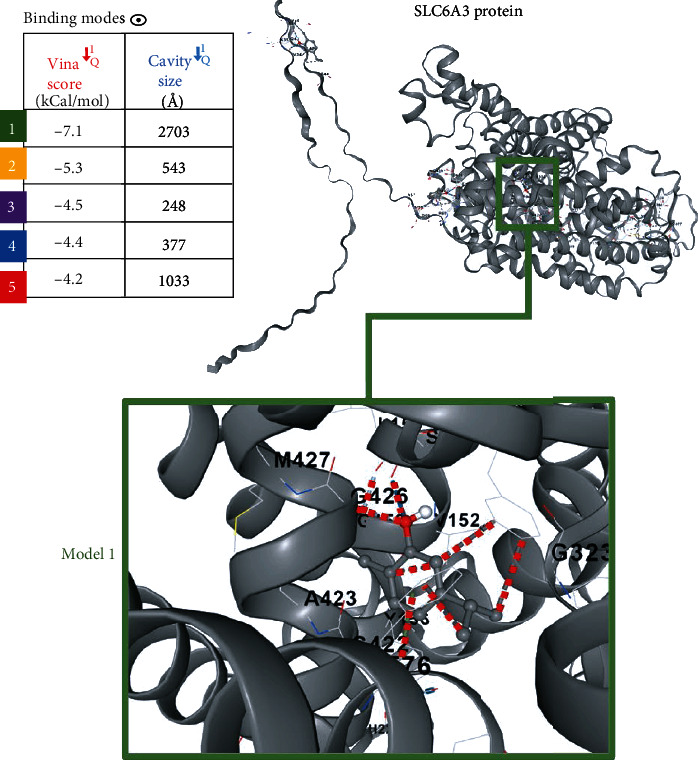
Cavity-detection guided blind docking models of the interaction of Carvacrol and SLC6A3 protein.

As we suggested Carvacrol might exert a direct effect on SLC6A3, we established cavity-detection guided blind docking models of the interaction of Carvacrol and SLC6A3 protein using the CB-Dock. The structure used in the docking model was from the Pubchem and the AlphaFold. The docking predicted five potential biding configurations of the interaction of Carvacrol and SLC6A3 protein, with vina scores of -7.1, -5.3, -4.5, -4.4, and -4.2 kCal/mol, respectively (Figure 7). Vina scores of -10 or lower usually represent a very good binding, and scores of -7 to -10 kCal/mol might be considered good binding. Only one of our models passed the cutoff score of -7 kCal/mol, therefore, we suggested model one was the most likely binding configuration.

### 2.9. The Regulation of SLC6A3 in the Viability of LIHC

**Figure 8:**
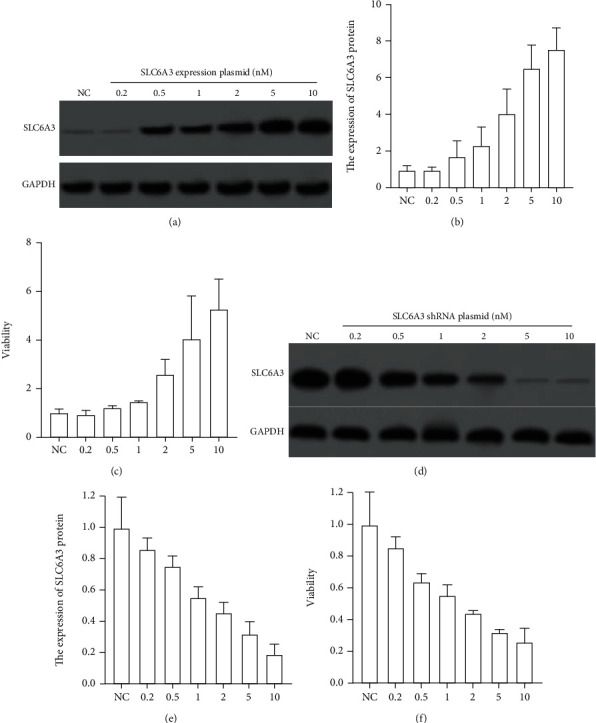
Validation of the essentials of SLC6A3 in the viability of LIHC cell line SNU-449. (a) Image of the western blotting of the protein expression of SLC6A3 in SNU-449 with different levels of SLC6A3 overexpression. (b) The protein expression of SLC6A3 in SNU-449 with different levels of SLC6A3 overexpression. (c) The cell viability of SNU-449 after 24-hour exposure to 200 *μ*M Carvacrol with different levels of SLC6A3 overexpression. (d) Image of the western blotting of the protein expression of SLC6A3 in SNU-449 with different levels of SLC6A3 knockdown. (e) The protein expression of SLC6A3 in SNU-449 with different levels of SLC6A3 knockdown. (f) The cell viability of SNU-449 after 24-hour exposure to 200 *μ*M Carvacrol with different levels of SLC6A3 knockdown.

To validate the regulatory effect of SLC6A3 on the viability of LIHC cells, we conducted SLC6A3 overexpression and knockdown experiments in a LIHC cell line SNU-449 and determined their effect on the cell viability. SNU-449 had a medium expression of SLC6A3, and it showed a point that was the closest to the linear regression model of viability and SLC6A3 level (Figure 5(e)), thus, we think it might be the best cell line to demonstrate the role of SLC6A3 in viability. Results showed that 0.2-10 nM of SLC6A3 expressing plasmid concentration-dependently improved the levels of SLC6A3 in SNU-449 cells (Figures 8(a) and 8(b)). The MTT assay revealed that the overexpression SLC6A3-dependently increased the viability of cells (Figure 8(c)). In addition, we also knocked down SLC6A3 expression in SNU-449. Results showed that 0.2-10 nM of SLC6A3 shRNA plasmid concentration-dependently reduced the levels of SLC6A3 in SNU-449 cells (Figures 8(a) and 8(b)). The MTT assay showed that the knockdown SLC6A3-dependently decreased the viability of cells (Figure 8(c)). These results indicated that SLC6A3 positively regulated the viability of LIHC cell line SNU-449.

## 3. Discussion

A previous study has reported the potential preventive effect of Carvacrol against diethylnitrosamine-induced LIHC in rats [37]. In our study, data supported that Carvacrol inhibited the viability of multiple LIHC cell lines which might account for the preventive effect of Carvacrol against LIHC in rats. In breast cancer cells, Carvacrol at 50-500 *μ*M significantly inhibited cell viability. For LIHC cells, our data revealed a similar effective concentration range at 10-300 *μ*M. In addition, Carvacrol at 100-600 *μ*M has been found to inhibit prostate cancer [22], and a higher concentration at over 500 *μ*M was required to significantly inhibit the viability of cervical tumor cell HeLa [21]. Based on these results, we suggested that different cancer types might have different sensitivity to Carvacrol.

However, whether Carvacrol has common targets among these cancer types remains unknown. In this study, we design a novel target screening study for Carvacrol in LIHC, which can also be used for other cancer types or even pan-cancer studies. BATMAN-TCM used a similarity-based method to predict potential targets of Carvacrol, the core idea of which was to rank potential drug-target interactions based on their similarity to the known drug-target interactions [34]. In this study, we obtained 40 target genes, among which, a large group of them were gamma-aminobutyric acids associated. Some of them might potentially interact with ion channels that affect cancers, such as two-pore channels [38]. Furthermore, many of these are also potential targets of anesthetic agents. Studies have suggested that anesthetics might potentially affect cancers [39–43], thus, Carvacrol might have actions to these effects.

We calculated the survival association of these target genes because we wanted to obtain potential targets that affect survival. So far, TCGA data were widely used in prognostic studies [44–46]. The potential association of gene expression and overall survival might identify biomarkers for cancer prognosis or functional cancer regulators for cancer. In this study, we used the survival association analysis to screen the potential functional molecule among the Carvacrol target genes. Eight target genes were identified including DRD1 and SCN4A, GABRA3, SLC6A3, GABRQ, PDE4D, GABRG3, and ALOX5. DRD1 was a gene associated with breast cancer [47] and lung cancer [48, 49]. SCN4A was sodium channel genes that might also affect cancer cells [50]. But the protective effect of DRD1 and SCN4A against LIHC has not been reported. In addition, GABA-associated genes (GABRA3, GABRQ, and GABRG3) are most expressed and function in the neurotransmitter in the mammalian brain. SLC6A3 is a dopamine transporter that is a member of the sodium- and chloride-dependent neurotransmitter transporter family [51]. Another target gene PDE4D was found functioning in colon cancer [52] and bladder cancer [53]. The last target gene, ALOX5, was reported to play a role in colon cancer [54], breast cancer [55], and lung cancer [56]. So far, these genes have not been studied in LIHC.

Among these 8 genes, we identified four genes that were expressed differently in LIHC and normal liver tissue. We suggested that the expression difference between cancer and noncancer tissue might indicate the potential mediation of this target for the cancer specificity effect of Carvacrol on LIHC treatments. Our results also found that Carvacrol can affect the expression of SLC6A3 in both LIHC cell lines and LIHC in patients. Clinical samples of this study were taken from liver cancer patients who were on separate medications, which weaken the consistency of the objects. We admitted that the doses were not chosen based on any standard criteria for bodyweight or medicine they were taking. The criteria we used to prescript was based on traditional Chinese medicine (TCM) theory which is difficult to descript in the paper. Technically, there was no preference for which patient used TCM or not because TCM is not necessary for the treatment according to the clinical instruction. Carvacrol was applied as a supplementary therapy for patients. Nevertheless, although not necessarily every one of them, it was clear that some of the patients with Carvacrol were significantly reduced in SLC6A3. We suggested the inconsistency of the medication might account for the inconsistency in the decrease of SLC6A3 in the Carvacrol treated group. More systematic evidence from clinical trials is required in the future.

Knockdown and overexpression experiment further confirmed that SLC6A3 was a biomolecule that promotes the viability of LIHC cells. Therefore, our data suggested that SLC6A3 mediated the inhibition of Carvacrol on the viability of LIHC cells. SLC6A3 has previously been reported as a potential circulating biomarker for gastric cancer detection and progression monitoring [57]. In addition, SLC6A3 was also found overexpressed and functioning in kidney cancer [58] and was suggested as a biomarker for patients with renal cell carcinoma [59]. However, to date, the role of SLC6A3 in LIHC has not been reported. In this study, we were the first to report the promotion effect of SLC6A3 on LIHC. We also predicted the binding conformation of the interaction between Carvacrol and SLC6A3 protein. Animal *in vivo* models have been widely used for medical study [8, 60], we hope the conclusion can be further validated with *in vivo* experimental evidence in the future. In addition, an alternative therapeutic method for LIHC, targeting cancer stem cells, has been proposed as a promising approach [61]. As Carvacrol affected the viability of LIHC cells, we proposed that its effect might be mediated by cancer stem cells.

To conclude, in the present study, we screened pharmacological targets of Carvacrol in LIHC. We identified SLC6A3 as a potential target of Carvacrol. This study is conducive to the optimization of the clinical application of Carvacrol in LIHC treatment.

## 4. Methods

### 4.1. Bioinformatic Analysis

BATMAN-TCM [62] was used to predict potential targets of Carvacrol. The LIHC TCGA mRNA-seq data with clinical information were accessed from The Cancer Genome Atlas (TCGA) [63] in January 2020. R foundation for statistical computing (2020) version 4.0.3 and ggplot2 [64] [65, 66](v3.3.2) was used to conduced bioinformatic analysis. The structure file of Carvacrol was downloaded from Pubchem [67]. The protein structure of SLC6A3 was predicted by AlphaFold [68], a state-of-the-art AI system developed by DeepMind. Cavity-detection-guided blind docking models of the interaction of Carvacrol and SLC6A3 protein were conducted using the CB-Dock [69].

### 4.2. The Collection of LIHC Tissues

LIHC tissues were collected from 36 patients with surgical treatment or biopsy including 18 patients with Carvacrol treatment and 18 patients without Carvacrol treatment. The prescription of Carvacrol treatment depended on the clinical condition of the patients, and the Carvacrol was applied as a supplementary therapy for patients. Samples were fixed, embedded in paraffin, and stored in 4°C. All donors were over 18 years old and have given formal consent to the use of their samples. The study has been approved by the Ethics Committee of the Tumor Hospital Affiliated To Nantong University (no. 2020-082).

### 4.3. Cell Culture

SNU-182, SNU-398, SNU-449, SK-HEP-1, HEP-3B2.1-7, SNU-387, PLC/PRF/5, Hep-G2, MEF, and THLE-2 were from ATCC (Washington, USA). All cells were cultured in DMEM medium with 10% Foetal Bovine Serum (FBS) in an incubator of 5% CO_2_ and 37°C.

### 4.4. Plasmid Transfection

SLC6A3 knockdown and overexpression were achieved by transfecting SLC6A3 shRNA plasmid or SLC6A3 expression plasmid into cells. The predesigned SLC6A3 expression plasmids (pDONR221_SLC6A3, Plasmid #132160) were purchased from the Addgene (Watertown, MA, USA). Human SLC6A3 shRNA silencing Adenovirus plasmids (Ad-h-SLC6A3-shRNA and shADV-223569) were purchased from the VECTOR BIOLAB (Malvern, PA, USA). Scrambled shRNA Control plasmid, and expression control plasmid was provided from the same source. Lipofectamine® 2000 was used to conduct the experiments following the instruction. In detail, seed cells at 70–90% confluency at transfection. Dilute four amounts of Lipofectamine® Reagent in Opti-MEM® Medium. Dilute DNA in Opti-MEM® Medium. Add diluted DNA to diluted Lipofectamine® 2000 Reagent (1 : 1 ratio). Incubate for 5 minutes at room temperature. Add DNA-lipid complex to cells. Incubate cells for 1–3 days at 37°C. Then, analyze transfected cells.

### 4.5. Drug

Carvacrol was purchased from Sigma-Aldrich. The dimethyl sulfoxide (DMSO) 1% was used as an emulsifier. DMSO (1%) in water was used as the negative control.

### 4.6. QPCR

The mRNA expressions were determined using a QPCR assay [70]. RNA was extracted using the RNeasy Mini kit (Qiagen, Germantown, MD, USA). The PrimeScript RT Reagent kit with gDNA Eraser (Takara Bio, Japan) and the PowerUp™ SYBR™ Green Master Mix (Thermo, Beverly, MA, USA) was used to conduct the retro transcription and QPCR. The Applied Biosystems StepOnePlus instrument (Thermo, Beverly, MA, USA) was used to run all the reactions. The results were normalized using the 2^-*ΔΔ*CT^ method.

Primers:

GABRA3 forward: 5′-CATTCATCCTTCTCTCCTTTCC-3′

GABRA3 reverse: 5′-GTTCTTGTCGTCTTGATTCCC-3′

GABRQ forward: 5′-CCCCACCTCTGTTCCTTTTC-3′

GABRQ reverse: 5′-CAGCACCCTGTCCAAAATC-3′

SCN4A forward: 5′-TCTTCCACTCCTTCCTCATC-3′

SCN4A reverse: 5′-TCATCTCGCCATCCTCATC-3′

SLC6A3 forward: 5′-TCACCACCTCCATCAACTCC-3′

SLC6A3 reverse: 5′-TCACTGACTCCATACCACCC-3′

GAPDH forward 5′-GAAGGTGAAGGTCGGAGTC-3′

GAPDH reverse: 5′-GAAGATGGTGATGGGATTTC-3′

### 4.7. Western Blotting

The protein expression of SCN4A and SLC6A3 was analyzed in western blotting experiments. The protein lysing buffer (Pierce, Rockford, IL, USA) with protease inhibitors (Roche, Indianapolis, IN, USA) was used to isolate proteins in samples. These proteins were separated in premade10–12% SDS-PAGE gels. These proteins were then transferred to 0.45 *μ*m PVDF membranes. The membrane was blocked in the western blotting blocking buffer. Then, the membranes were incubated with the primary antibodies (Polyclonal Rabbit anti-Human SCN4A Antibody LS-C200644, Human/Primate SLC6A3/DAT1 Extracellular Loop 2 Antibody PPS069, and Anti-GAPDH Antibody G-9 sc-365062) overnight at 4°C and secondary antibodies (mouse anti-rabbit IgG-HRP: sc-2357) at RT for 1 hour. ECL solution was used to visualize the protein on the membrane.

### 4.8. Immunochemistry Staining

SLC6A3 staining was done by immunochemistry using SLC6A3/DAT1 Antibody NBP2-68583 (Centennial, CO, USA). Briefly, paraffin-embedded tissue samples were deparaffinized in xylene, rehydrated through graded ethanols, and then submerged into the citric acid buffer for heat-induced antigenic retrieval, blocked with 10% bovine serum albumin, incubated with SLC6A3 primary antibodies at 4°C overnight, and developed using the DAKO ChemMate Envision Kit HRP (Dako-Cytomation, Carpinteria, CA, USA) followed by counterstaining with hematoxylin, dehydration, clearing, and mounting.

### 4.9. Viability Assay

The cell viability was determined using the MTT assay [71]. The cells were plated in 96-well plates (3–5 × 103/well) for 12 h for adhesion and exposed to 200 *μ*M Carvacrol for 24 hours. Then, cells were incubated with 20 *μ*L of 5 mg/mL MTT (Abcam, Cambridge, UK) for 2 h, and the resulting formazan crystals were dissolved in 200 *μ*L DMSO. The A490 was measured using the Thermo Scientific™ Multiskan™ (Waltham, MA, USA) FC Microplate Photometer. All data were normalized to the vehicle control or the negative control (NC) group.

### 4.10. Statistical Analysis

The experiment was performed at least in triplicate and repeated three independent times. Data were presented in means ± standard deviations in the bar charts. A *t*-test or ANOVA was used to assess the significance (*p* < 0.05). Dunnett's post hoc tests were used to test the difference between groups. The GraphPad Prism (version 8) was used to calculate statistics.

## Figures and Tables

**Table 1:** Viability IC50 values of Carvacrol.

Cell line	Cancer or primary	Viability IC50 values of Carvacrol (*μ*M)
SNU-182	Cancer	68.8
SNU-398	Cancer	36.3
SNU-449	Cancer	48.6
SK-HEP-1	Cancer	89.9
HEP-3B2.1-7	Cancer	81.1
SNU-387	Cancer	49.3
PLC/PRF/5	Cancer	41.2
Hep-G2	Cancer	24.2
MEF	Primary	>300
THLE-2	Primary	>300

## Data Availability

The data that support the findings of this study are available from the corresponding author upon reasonable request.

## Supplementary Materials

Supplementary Materials The supplementary file contains the graphical abstract.
