# Supplementary figures and images for: Screening and Validation of a Carvacrol-Targeting Viability-Regulating Protein, SLC6A3, in Liver Hepatocellular Carcinoma
Source: Dis Markers. 2022 Mar 30;2022:3736104. doi: 10.1155/2022/3736104 (PMC8986433; doi:10.1155/2022/3736104)

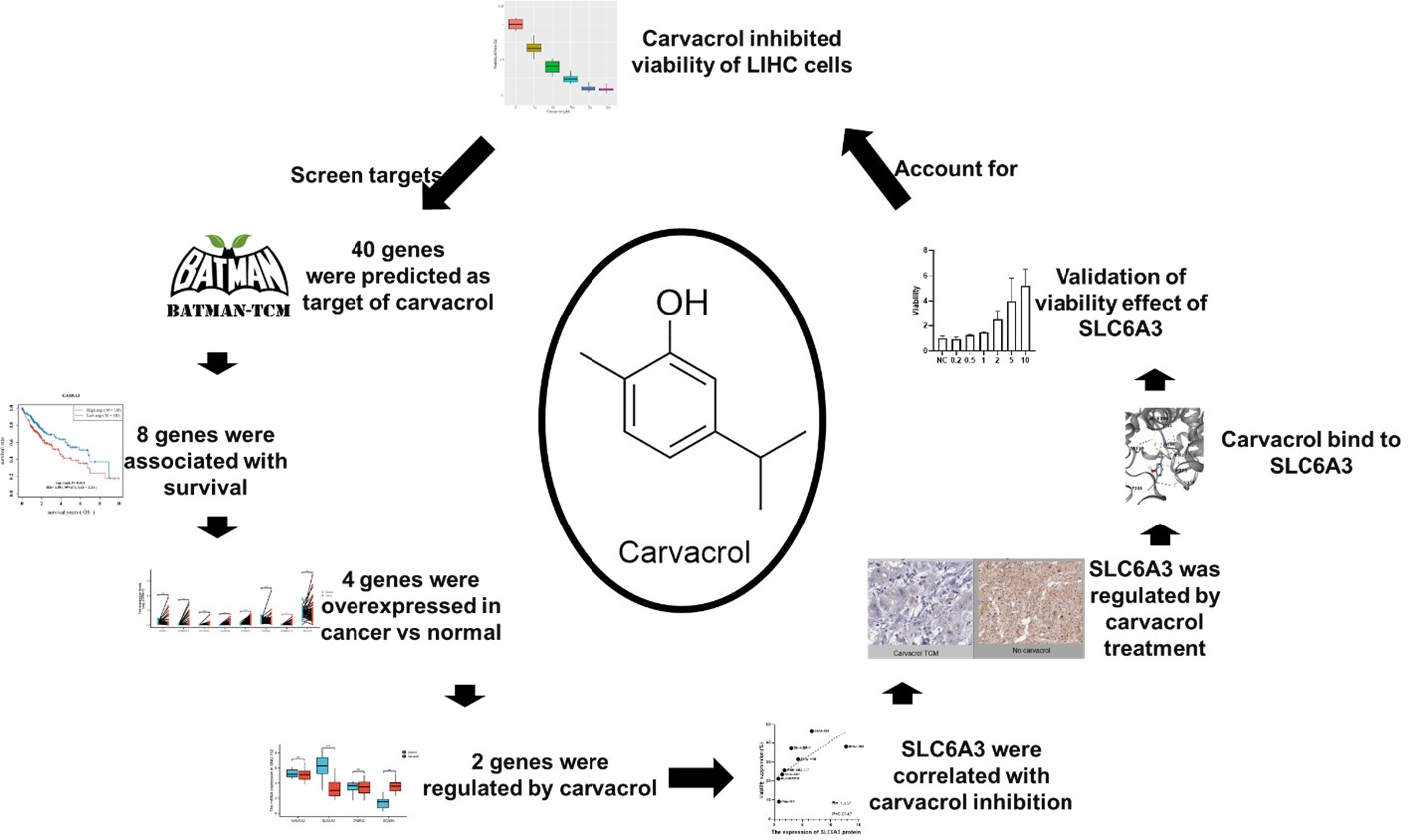

Supplement: Supplementary Materials — The supplementary file contains the graphical abstract. [file 3736104.f1.jpg]
